# Pathogenic CANVAS-causing but not nonpathogenic *RFC1* DNA/RNA repeat motifs form quadruplex or triplex structures

**DOI:** 10.1016/j.jbc.2023.105202

**Published:** 2023-09-01

**Authors:** Mohammad Hossein Abdi, Bita Zamiri, Gholamreza Pazuki, Soroush Sardari, Christopher E. Pearson

**Affiliations:** 1 Department of Chemical Engineering, Amirkabir University of Technology (Tehran Polytechnic), Tehran, Iran; 2 Drug Design and Bioinformatics Unit, Department of Medical Biotechnology, Pasteur Institute of Iran, Tehran, Iran; 3 Program of Molecular Genetics, University of Toronto, Toronto, Ontario, Canada; 4 Program of Genetics & Genome Biology, The Hospital for Sick Children, The Peter Gilgan Centre for Research and Learning, Toronto, Ontario, Canada

**Keywords:** RNA, DNA, G-quadruplex, triplex DNA, CANVAS, *RFC1*, ataxia, spectroscopy

## Abstract

Biallelic expansions of various tandem repeat sequence motifs are possible in *RFC1* (replication factor C subunit 1), encoding the DNA replication/repair protein RFC1, yet only certain repeat motifs cause cerebellar ataxia, neuropathy, and vestibular areflexia syndrome (CANVAS). CANVAS presents enigmatic puzzles: The pathogenic path for CANVAS neither is known nor is it understood why some, but not all expanded, motifs are pathogenic. The most common pathogenic repeat is (AAGGG)n•(CCCTT)n, whereas (AAAAG)n•(CTTTT)n is the most common nonpathogenic motif. While both intronic motifs can be expanded and transcribed, only r(AAGGG)n is retained in the mutant *RFC1* transcript. We show that only the pathogenic forms unusual nucleic acid structures. Specifically, DNA and RNA of the pathogenic d(AAGGG)4 and r(AAGGG)4 form G-quadruplexes in potassium solution. Nonpathogenic repeats did not form G-quadruplexes. Triple-stranded structures are formed by the pathogenic motifs but not by the nonpathogenic motifs. G- and C-richness of the pathogenic strands favor formation of G•G•G•G-tetrads and protonated C+-G Hoogsteen base pairings, involved in quadruplex and triplex structures, respectively, stabilized by increased hydrogen bonds and pi-stacking interactions relative to A-T Hoogsteen pairs that could form by the nonpathogenic motif. The ligand, TMPyP4, binds the pathogenic quadruplexes. Formation of quadruplexes and triplexes by pathogenic repeats supports toxic-DNA and toxic-RNA modes of pathogenesis at the *RFC1* gene and the *RFC1* transcript. Our findings with short repeats provide insights into the disease specificity of pathogenic repeat motif sequences and reveal nucleic acid structural features that may be pathogenically involved and targeted therapeutically.

Cerebellar ataxia with neuropathy and vestibular areflexia syndrome (CANVAS) is a late-onset progressive neurodegenerative disease (1). Large biallelic repeat expansions of 250 to >2000 repeats in intron 2 of the replication factor C subunit 1 (*RFC1*) gene have been identified as the cause for disease (Fig. 1*A*) (2, 3, 4). A refractory chronic cough typically precedes neurological symptoms by >20 years and is present in monoallelic *RFC1*-expansion carriers (5, 6). Five different pathogenic expanded repeat motifs have been observed; with the AAGGG repeat having the highest frequency in patients (Fig. 1*A*) (2, 3). The nonpathogenic motifs contained AAAAG or AAAGG expanded repeats, with the former reported as the most common nonpathogenic allele (3, 4). Both nonpathogenic and pathogenic repeats can be massively expanded. It is not clear why some expanded motifs cause disease and other expanded motifs do not. The motif sequences suggest that higher-order nucleic acid structures might be involved and that pathogenic motifs are forming structures that nonpathogenic motifs are not.

**Figure 1:**
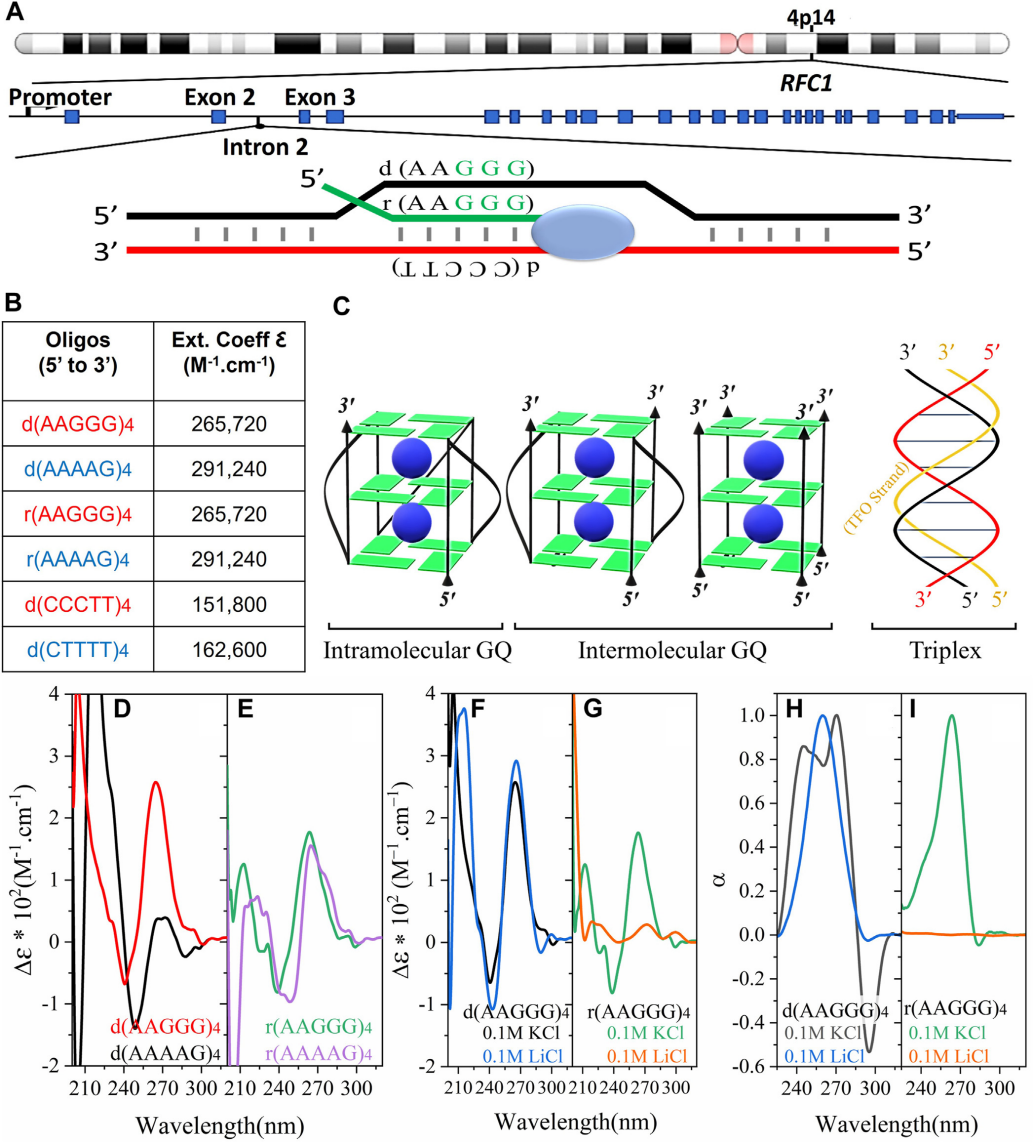
**Pathogenic but not nonpathogenic DNA and RNA repeats form quadruplex structures.***A*, pathogenic and nonpathogenic expanded repeat motifs identified in the transcribed intron 2 of the *RFC1* gene where the pathogenic repeat is retained, whereas the nonpathogenic repeat is excised by splicing mechanisms (3). Both are translated to the full-length RFC1 protein. *B*, sequences of the ribo-oligonucleotides and deoxyribo-oligonucleotides used here in *red*- and *blue*-fonted oligos are pathogenic and nonpathogenic, respectively. *C*, schematic of intramolecular and intermolecular G-quadruplex DNA structures consisting of three G-quartets form and schematic of a triplex DNA structure. *D*, CD spectra of 5 μM of DNA repeats. *E*, RNA repeats of pathogenic (AAGGG)4 and nonpathogenic (AAAAG)4 repeats. *F* and *G*, the effect of lithium ions on CD spectra. *H* and *I*, normalized thermal difference spectra (TDS) of 5 μM d(AAGGG)4 and *r(AAGGG)4* in 10 mM Tris and 0.1 mM EDTA (pH 7.4) at 25 °C. RFC1, replication factor C subunit 1.

The exact etiology and pathogenesis of CANVAS is puzzlingly unclear. Recent data support a loss-of-function mechanism caused by the homozygous *RFC1* pathogenic repeat expansion motifs (7). The RFC1 protein is part of the pentameric complex of RFC (replication factor C) that is involved in DNA replication and repair.

Homopurine sequences (guanines and adenines) and homopyrimidine (cytosines and thymines) DNA and RNA strands can self-associate or interassociate to form a wide variety of non-B-form structures. These structures can be biophysically polymorphic and involve different purine–purine base pairs, such as G•G, A•A, and G•A, which are the basis for multiple folding patterns (8). For instance, d(GA)n has been shown to self-associate into parallel and antiparallel homoduplexes, quadruplexes, and triplexes (9, 10). Expansions of d(GAA)n•(TTC)n, associated with Friedreich’s ataxia, were shown to form higher-order structures revealed to be involved in disease (11, 12, 13). Environmental conditions including counterions, pH, and temperature can influence and determine the conformational polymorphism.

High guanine content in homopurine tracts under certain environmental conditions can assume quadruplex structures (Fig. 1*C*) (10). G-quartets are the building blocks for the four-stranded quadruplex structures. G-quartets are planar arrangements of four guanine molecules self-associated *via* Hoogsteen hydrogen bonds around a monovalent cation such as potassium or sodium. G-quartets stack on top of each other, stabilized by pi–pi interactions to form an overall helical arrangement. They can form from a single strand or multiple strands (14, 15). Quadruplexes have been linked to various biological processes, including genomic instability, gene regulation, transcript splicing, and RNA translation regulation (16, 17, 18).

The presence of a homopurine•homopyrimdine duplex and a single-stranded purine or pyrimidine strand can form triple-stranded DNAs (Fig. 1*C*). In triplexes, the third strand (*italicized*) is bound to the major groove of the complementary stranded double helix (indicated by “•”) *via* alternative hydrogen bonding (Hoogsteen base pairing, indicated by a “-”) resulting in C•G-*C*^*+*^ and T•A-*T* triads where in most cases, cytosines require protonation (indicated by “+”) to bind to the major groove. As previously reviewed, neurons under stressed conditions such as neurodegeneration can experience intranuclear imbalances of pH often toward acidic levels (pH < 7) (19, 20). Cytosine residues become protonated under slightly acidic pH (pH 4.5–6.2), favoring triplex formation (21).

Here, we report the formation of G-quadruplexes and triplexes in the pathogenic motif d(AAGGG)4 but not in the nonpathogenic d(AAAAG)4. Quadruplex formation is also confirmed in the pathogenic RNA r(AAAGGG)4 but not in the nonpathogenic repeat r(AAAAG)4. We also reveal triple-stranded structures formed by the pathogenic (AAGGG)4•(CCCTT)4-*(CCCTT)4* motifs but not by the nonpathogenic motif. Our findings support the concept that these unusual structures may participate in disease pathogenesis.

## Results

### Quadruplex structure formation by DNA/RNA of pathogenic repeats (CD)

Since the composition of the pathogenic repeats (AAGGG)n•(CCCTT)n are both homopurine/homopyrimidine mirror repeats (22) and contain evenly spaced G3 runs (23), we hypothesized these repeats could assume triplex and/or quadruplex structures (Fig. 1, *A*–*C*). Previous studies revealed that a minimum of two to four contiguous repeat units was sufficient to detect triplex and quadruplex structures by GAA/TTC and CGG/CCG repeats (24, 25, 26, 27). Therefore, we used CD spectroscopy to investigate the aforementioned and study the structural differences between pathogenic (AAGGG)4 and nonpathogenic (AAAAG)4 DNA and RNA repeats at physiological conditions. As seen in (Fig. 1*D*), the CD spectra of d(AAGGG)4 in KCl (pH 7.4) exhibits positive peaks around 265 and 210 nm with a negative peak around 240 nm, the signature spectra of parallel stranded G-quadruplexes (28). The nonpathogenic d(AAAAG)4 has a positive low-intensity peak around 270 nm and a high-intensity peak around 215 nm with negative peaks around 250 and 205 nm. For RNA (Fig. 1*E*), the pathogenic r(AAGGG)4 exhibits positive peaks around 265 nm and a negative peak around 240 nm; a signature of parallel-stranded RNA G-quadruplexes, whereas the nonpathogenic variant r(AAAAG)4 has positive peaks around 265 nm and 225 nm alongside a shoulder around 280 nm with negative peaks around 245 and 210 nm. A positive 260 nm peak and a negative 210 nm have been previously associated with A-form duplex formation in RNA molecules (28). Accordingly, potassium-induced G-quadruplex formation in both pathogenic DNA and RNA repeats of (AAGGG)4 is seen. In order to further confirm this, the effect of changing environmental conditions such as cation type has been studied.

G-quadruplex formation is largely cation dependent, and Li^+^ ions are known to destabilize them. To show this, CD spectra (Fig. 1, *F* and *G*) and thermal difference spectra (TDS) (Fig. 1, *H* and *I*) of (AAGGG)4 DNA and RNA repeats in 100 mM LiCl were recorded. In Figure 1*F*, d(AAGGG)4 exhibits a positive peak at 265 nm and a negative peak at 240 nm (signature G-quadruplex signals) in both K^+^ (*black*) and Li^+^ (*blue*). In Li^+^, negative peaks at 215 and 290 nm are seen. CD signals around 265, 250, and 215 nm have been attributed to homoduplexes (8). In Figure 1*G*, the effect of Li^+^ on the CD spectra of r(AAGGG)4 is more pronounced and the CD signals at 260 and 240 nm disappear in Li^+^. The TDS is the difference in the UV spectra of folded *versus* unfolded structures and was shown to provide a distinctive signature for nucleic acid structures (29). In Figure 1, *H* and *I*, TDS of d(AAGGG)4 and r(AAGGG)4 in K^+^ and Li^+^ are not superimposed. For d(AAGGG)4 in Li^+^, the TDS exhibits a 257 nm positive peak with a minor negative peak around 293 nm, associated with GA duplex formation (29). In K^+^, positive peaks at 243 and 273 nm and an intense negative peak at 295 nm confirm G-quadruplex formation. For r(AAGGG)4, consistent with the CD results, lithium was shown to destabilize the RNA higher-order structures according to the TDS as well (Fig. 1*I*). Thus, potassium-induced G-quadruplex formation in pathogenic DNA and RNA repeats is further confirmed. Next, the thermal stability of the repeats in K^+^ *versus* Li^+^ is studied.

### Structure formation by DNA/RNA of pathogenic repeats (melting)

**Figure 2:**
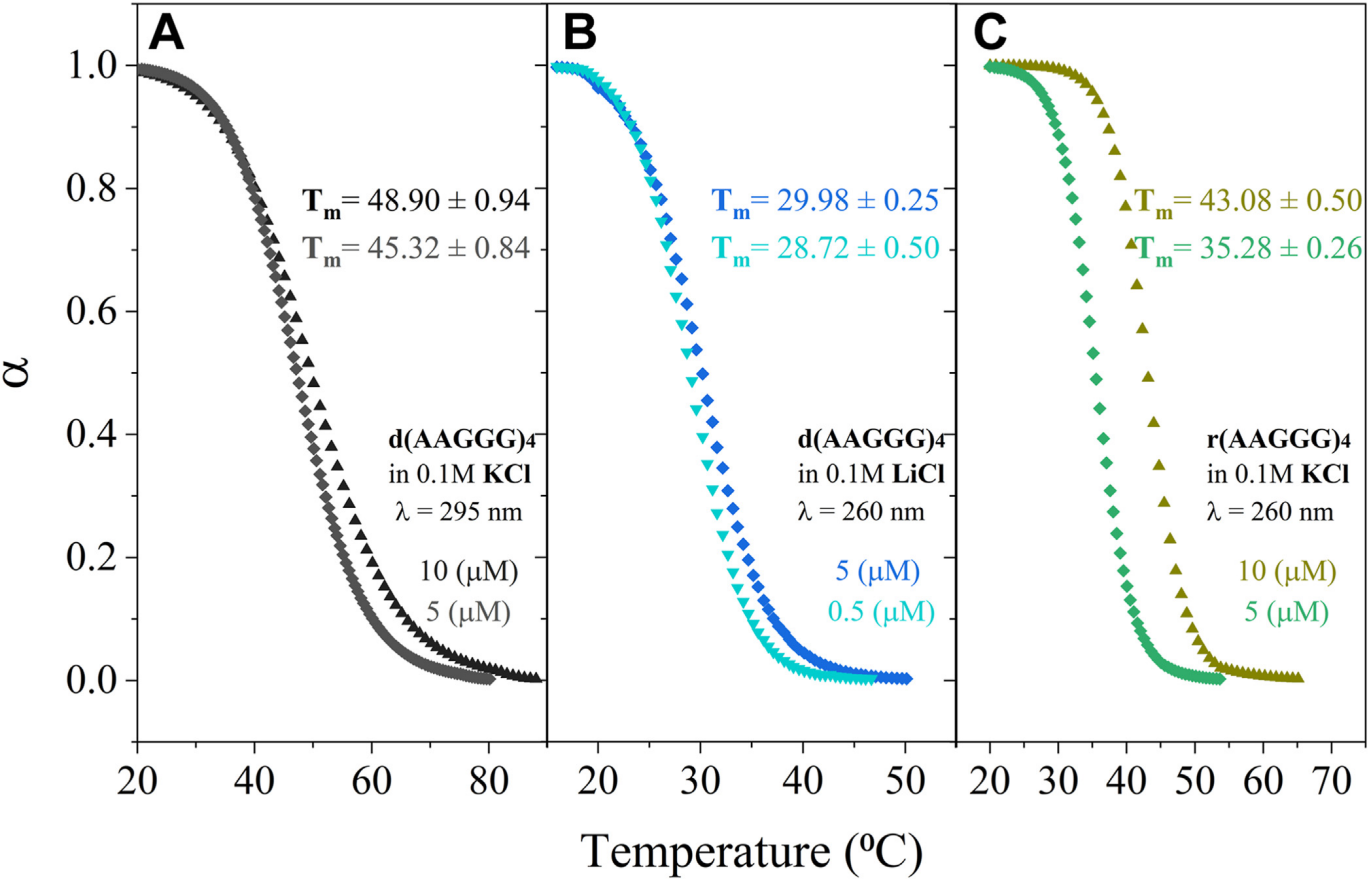
**Effect of LiCl and strand concentration on the normalized UV thermal melting profiles.***A*, d(AAGGG)4 in 0.1 M KCl. *B*, d(AAGGG)4 in 0.1 M LiCl. *C*, r(AAGGG)4 in 0.1 M KCl in 10 mM Tris–HCl and 0.1 mM EDTA (pH 7.4) at different strand concentrations.

UV thermal melting profiles are used to gain structural insight by studying thermal stability in K^+^ *versus* Li^+^ and specify molecularity (based on the concentration dependence of *T*_m_) (30). In Figure 2*A*, upon doubling the concentration of d(AAGGG)4, the *T*_m_ has increased from 45.32 to 48.90 °C, showing multimolecularity. Also, *T*_m_ was shown to decrease from 45.32 °C in K^+^ to 32.17 °C in Li^+^ and was concentration independent (Fig. 2*B*). Also, in K^+^, thermal meltings were performed at 295 nm, a signature wavelength for recording G-quadruplex melting transitions, and in Li^+^, no transition was recorded at 295 nm (not shown). The RNA G-quadruplexes formed in K^+^ were shown to have a *T*_m_ of 35.28 ± 0.7 and 43.08 ± 0.5 at 5 μM and 10 μM strand concentrations, respectively, also showing multimolecularity (Fig. 2*C*), whereas in Li^+^, no melting phenomenon was observed (not shown). Therefore, potassium-induced multimolecular G-quadruplexes are shown for DNA and RNA of d(AAGGG)4 repeats. Consequently, the tendency of the pathogenic and nonpathogenic repeats to form triplex structures was studied using mixing curves.

### Strand proportions affect structure formation

**Figure 3:**
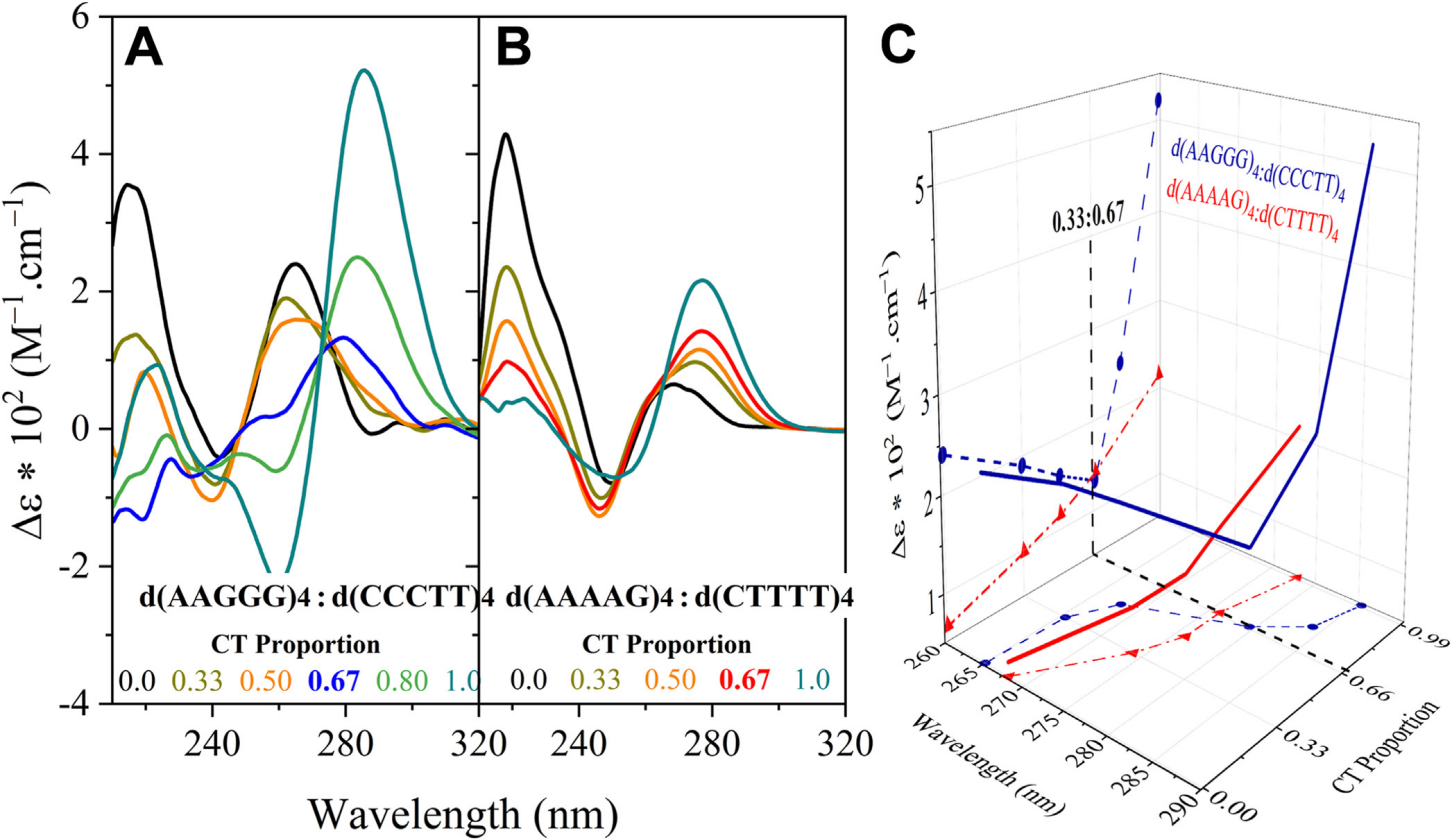
**Triplex formation by pathogenic but not nonpathogenic DNA repeats.** CD spectra of mixtures of (*A*) d(AAGGG)4:d(CCCTT)4 and (*B*) d(AAAAG)4:d(CTTTT)4 at the indicated molar portions; in 6 mM sodium phosphate buffer and 150 mM NaCl (pH 5.6) at 25 °C. *C*, effect of increasing the proportion of d(CCCTT)4 on the signature positive CD ellipticity of d(AAGGG)4•d(CCCTT)4 mixtures compared with the effect of parallel reactions of increasing the proportion of d(CTTTT)4 on the signature positive CD ellipticity of d(AAAAG)4•d(CTTTT)4 mixtures. Increased molar levels of d(CCCTT)4 relative to duplexes d(CCCTT)4•d(AAGGG)4 to 0.67:0.33 marks the maximum deviation in the CD spectrum of the d(TTCCC)4•d(AAGGG)4-*days(TTCCC)4* triplex from the weighted average of the spectra of the individual strands. A shift to a triplex signal was not observed for d(AAAAG)4•d(CTTTT)4) mixtures with increased proportions of d(CTTTT)4.

Mixing curves, resulting from recording the CD spectra of a mixture of predetermined proportions of d(AAGGG)4 and d(AAAAG)4 with their complementary strands, are used to demonstrate triplex formation in the pathogenic and nonpathogenic repeats (Fig. 3). The homopyrimidine d(CCCTT)4 exhibit a prominent positive peak around 285 nm and a negative peak at 260 nm; and d(CTTTT)4 a positive peak around 280 nm and a negative peak around 250 nm at pH 5.6, confirming *i*-motif tetraplex formation (21). In Figure 3*A* for pathogenic d(AAGGG)4, upon increasing the proportion of d(CCCTT)4 in the d(CCCTT)4:d(AAGGG)4, mixture, the maximum intensity positive peak of the CD spectra red shifts to higher wavelengths initially accompanied by a reduction in intensity followed by an increase in intensity. The change in this peak is shown in Figure 3*C* (wavelength *versus* CT proportion, *blue points*). The breakpoint in the mixing curve (Δε *versus* CT proportion, *blue points*) indicates d(CCCTT)4•d(AAGGG)4-*d(CCCTT)4* triplex formation at 0.67:0.33 M proportion of d(CCCTT)4:d(AAGGG)4. This was confirmed by plotting the deviation of the weighted average of the spectra of the individual strands at different molar proportions of d(CCCTT)4, with the observed spectra of the mixing curves at the same molar proportions; which is maximal at 0.67 M proportion. At this molar proportion, isodichroic points are seen at 272 and 239 nm (Fig. S1). In contrast, the mixing curve for the nonpathogenic d(AAAAG)4 shown in Figure 3*B*, show that upon increasing the proportion of d(CTTTT)4, the maximum positive peak of the CD spectra red shifts to higher wavelengths with an intensity increase (also seen in Figure 3*C*, wavelength *versus* CT proportion, *red points*). Unlike the pathogenic repeats, the weighted average of the spectra of the individual strands of d(CTTTT)4 and d(AAAAG)4 at 0.67 M proportion of d(CTTTT)4 is superimposed with the observed mixing curve spectra (Fig. S1). This along with the absence of a breakpoint in the Δε *versus* CT proportion in Figure 3*C* (*red points*) is indicative of the absence of triplex DNA formation for the nonpathogenic repeats at the same environmental conditions and points toward the d(AAAAG)4 and d(CTTTT)4 strands forming self-complexes separately. Also, no isodichroic points are observed.

### Both pH and counterions affect structure formation

**Figure 4:**
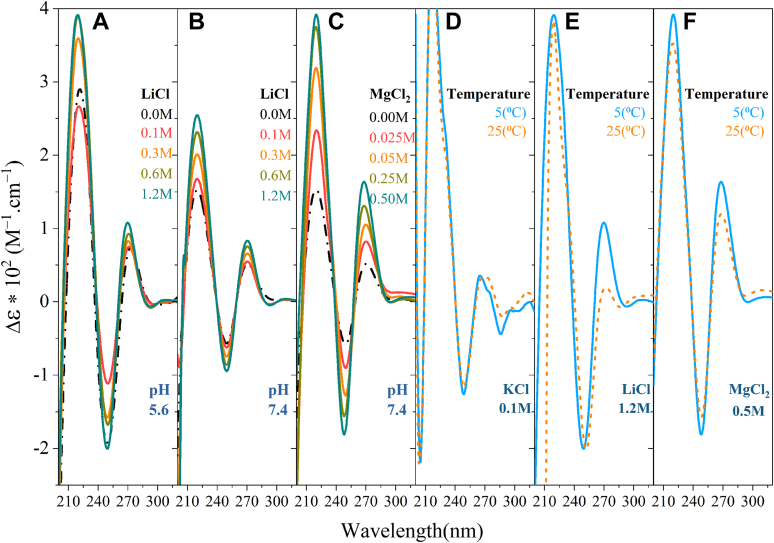
**Assessment of homoduplex formation in the nonpathogenic DNA repeats using CD spectra of 5 μM d(AAAAG)4**. *A*, indicated concentrations of LiCl in 6 mM potassium phosphate and 0.1 mM EDTA (acidic pH 5.6) at 5 °C. *B*, indicated concentrations of LiCl in 6 mM potassium phosphate and 0.1 mM EDTA (pH 7.4) at 5 °C. *C*, indicated concentrations of MgCl_2_ in 10 mM Tris–HCl and 0.1 mM EDTA (pH 7.4) at 5 °C. *D*, 10 mM Tris–HCl, 0.1 mM EDTA, 100 mM KCl (pH 7.4) at 5 and 25 °C. *E*, 6 mM potassium phosphate, 0.1 mM EDTA, 1.2 M LiCl (pH 7.4) at 5 and 25 °C. *F*, 10 mM Tris–HCl, 0.1 mM EDTA, and 50 mM MgCl_2_ (pH 7.4) at 5 and 25 °C.

Adenine-rich homopurine sequences such as d(AAAAG)4 can be biophysically polymorphic and largely dependent on environmental factors (8). Therefore, the effect of pH, temperature, and counterions has been studied (Fig. 4). In Figure 4, *A*–*C*, the effect of increasing Li^+^ at two different pHs and increasing Mg^2+^ at neutral pH is shown. At neutral pH (Fig. 4*B*), in the absence of Li^+^, the CD spectra exhibit a low-intensity positive peak around 270 nm, a negative peak around 250 nm, and a sharp positive peak around 220 nm. At acidic pH (Fig. 4*A*) and at 0 M LiCl, the 270 and 250 nm peaks are more pronounced. By increasing the ionic strength (Fig. 4, *A*–*C*), the 270 and 250 nm increase in intensity, showing secondary structure formation (21); consistent with reports in the literature that MgCl_2_ (31) and LiCl (8) promote homoduplex formation in homopurine tracts. In Figure 4, *D*–*F*, the effect of temperature is studied. At 0.1 M K^+^ (Fig. 4*D*), the 5 °C and 25 °C CD spectra are superimposed consistent with the absence of a melting transition (not shown), confirming a very low *T*_m_. In 1.2 M Li^+^ (Fig. 4*E*) and 0.5 M Mg^2+^, this was not the case, and lower temperatures increased the 270 nm peak. At lower concentrations of Li^+^, the CD spectra at two temperatures were superimposed (not shown). Furthermore, the TDS and melting transitions were recorded at 0.5 M MgCl_2_ and 1.2 M LiCl (Fig. S2) confirming secondary structure formation induced by increasing the ionic strength.

### Quadruplex ligand binds pathogenic repeats

**Figure 5:**
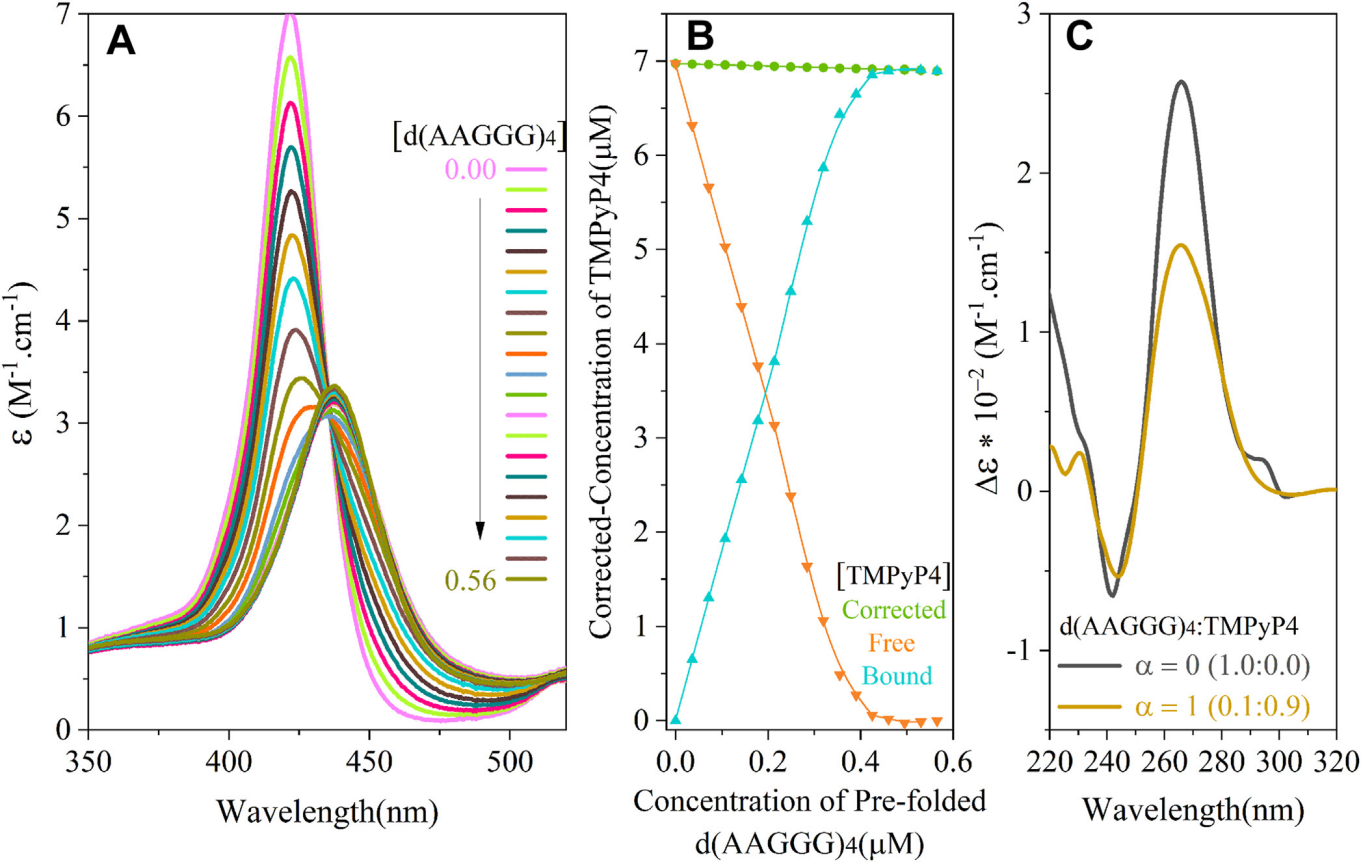
**TMPyP4 binds to pathogenic DNA repeats.***A*, visible absorption spectra of TMPyP4 in the absence and presence of an increasing concentration of prefolded d(AAGGG)4. *B*, free and bound TMPyP4 *versus* the concentration of prefolded d(AAGGG)4. *C*, CD spectra of d(AAGGG)4 with and without TMPyP4.

The interaction between TMPyP4 and G-quadruplexes formed by d(AAGGG)4 was studied by measuring the visible absorption spectra (Fig. 5). The d(AAGGG)4 oligonucleotides were titrated into a solution of TMPyP4, and the Soret band was monitored as a function of DNA concentration (Fig. 5*A*). The hypochromicity seen upon increasing concentrations of d(AAGGG)4 was 68% with a bathochromic shift of 16 nm indicative of the binding of TMPyP4 to quadruplexes formed by d(AAGGG)4. The data also reveal an apparent isosbestic point at 435 nm indicating the presence of two species. The effect of TMPyP4 on the folding pattern of d(AAGGG)4 is seen in Figure 5*B*, where at the molar proportion of TMPyP4:d(AAGGG)4 equal to 0.9 (where binding is complete), we see a 260 nm peak with lower intensity.

## Discussion

Homopurine sequences can adopt a variety of different folding patterns depending on the environmental conditions (8, 32). Here, we demonstrate that DNA and RNA of the pathogenic AAGGG but not the nonpathogenic AAAAG repeat motifs of *RFC1* form unusual structures whose formation is sensitive to pH, Li^+^, K^+^, and temperature. Quadruplexes form in DNA and RNA of the pathogenic repeat (AAGGG)4 in the presence of K^+^, exhibiting the signature CD signal (Fig. 1, *D* and *E*) and TDS (Fig. 1, *H* and *F*) of G-quadruplexes. In the presence of Li^+^, the DNA of the pathogenic repeats forms intramolecular foldbacks with lower stability through G•A and G•G pairs while preventing G-quadruplex formation (10). The similarity observed between the CD spectra of d(AAGGG)4 in K^+^ *versus* Li^+^ can been attributed to the fact that quadruplex formation can be the result of addition of two homoduplexes and that purine–purine stacking is conserved in the quadruplexes and intramolecular foldbacks (8). The DNA and RNA G-quadruplexes formed in K^+^ by the pathogenic (AAGGG)4 are multimolecular as their melting temperatures are concentration-dependent (33). As for the nonpathogenic d(AAAAG)4 repeats, the CD spectra at physiological conditions (Fig. 1*D*) with low amplitudes from 240 to 300 nm point toward a single-stranded conformer consistent with the literature (8). Lowering pH and increasing ionic strength promoted secondary conformers for d(AAAAG)4. The arising CD spectra at acidic pH and high ionic strengths has been previously associated with bimolecular duplexes in homopurine tracts (34, 35).

We have also revealed that at slightly low pH levels that can arise in stressed cells (19, 20), (CCCTT)4•d(AAGGG)4-*d(CCCTT)4* triplex structures are formed, whereas the nonpathogenic strands did not form triplexes in the presence of their complementary strands. Several factors including base composition, base sequence, expansion size, and environmental factors determine triplex stability (21, 36, 37, 38). For instance, the more G-rich the strand is, the more biophysically stable the triplex is. At low pH, C•G-*C*^*+*^ triads are more stable compared with T•A-*T* triads. While cytosine protonation permits G-C Hoogsteen pairs, protonation is not required for A-T Hoogsteen pairs in the T•A-*T* triads (39). Interrupting the G-richness with a single residue or multiple A residue amongst a run of G residues can diminish the propensity to form triplexes and their biophysical stability (37). Pi–pi stacking interactions of adjacent triads can dramatically affect stability of triplexes, G•G•G•G-quartets, or C•G-*C*^*+*^ triads and can favor the biophysical stability of G-quadruplexes or triplexes, respectively. Moreover, the sequence organization of a G+A repeat, including the mirror repeat of the sequence, extrusion point, and loop location, can dramatically affect triplex structures (40). Moreover, Hoogsteen base pairings, involved in both quadruplex and triplex structures, where these pairings can be biophysically stabilized by increased hydrogen-bond and pi-stacking interactions relative to the A-T Hoogsteen base pairs formed by the nonpathogenic (AAAAG)n•(CTTTT)n motif (41, 42).

The ability and propensity of the pathogenic but not nonpathogenic repeats to form unusual structures in the DNA and RNA suggests that such structures may participate in CANVAS pathogenesis. For example, the DNA structure formation may allow for larger expansion sizes, transmitted or somatic of the pathogenic *RFC1* repeats. CANVAS is caused by expansions of (AAGGG)250–2000 repeats. Our study has focused upon short tracts of RFC1 repeat motifs associated with CANVAS pathology and with neurotypically healthy individuals, we have not structurally assessed disease relevant lengths. While long tracts of the nonpathogenic motif, (AAAAG)n•(CTTTT)n, could form a triplex, its biophysical stability would likely be diminished relative to the expanded pathogenic motif, (AAGGG)n•(CCCTT)n, as A-tracts have been shown to disfavor triplexes (43). Similarly, the propensity to form a quadruplex by long tracts of r(AAAAG)n would be severely diminished relative to long tracts of r(AAGGG)n (23). While it is likely that longer tracts will have at least the same, if not greater propensity to form the unusual structures we demonstrate herein, we make no claim as to a length-dependent effect. We note that numerous studies have used only short nondiseased lengths of repeat tracts to biophysically reveal formation of higher order structures. Those initial studies revealed key structural features in subsequent studies of disease-relevant lengths. For example, intrastrand hairpins are critical features of slipped-DNAs assumed by diseased-length repeat tracts in patient tissues (44, 45, 46, 47). Similarly, triplexes and quadruplexes initially observed in short tracts were evident in diseased tracts in patient cells (24, 27, 48, 49), and in premutation/protomutation lengths, respectively (25, 26, 50, 51, 52). The (TTTCC)48•(GGAAA)48, of the quail *T64* gene, having a length shorter than *RFC1* CANVAS disease lengths, could form both PU-PY-PY and PU-PU-PY triplexes (53, 54). In the RNA, structure formation may contribute to the escape of repeat-containing intron excision, as many disease-associated G+C-rich repeats are retained in toxic RNAs (55). We use the term “toxic-DNA” much as the term “toxic-RNA,” wherein an unusual DNA sequence can be the source of toxicity either as direct source of toxicity, in that it is mutagenic or indirect by being the template for toxic-RNAs. The involvement of RNA secondary structure of the actively or newly produced transcripts, including those with G/A-rich homopurine motifs on alternative splicing of pre-mRNA has been reviewed (56, 57, 58, 59, 60). Such structural changes can occur in the DNA and RNA. The subcellular localization and processing of the mutant *RFC1* transcript may be altered. A loss-of-function pathway may arise through the sequestration of proteins bound to the expanded repeat motif and/or to quadruplex or triplex structures formed by the pathogenic alleles. As in other repeat diseases, such sequestered proteins could include those involved in a broad range of functions, such as transcription, RNA metabolism, chromatin regulation, and DNA repair (61, 62, 63).

The possible involvement of higher-order nucleic acid structures in disease has led to chemical targeting of these using small molecules. Ligands that bind to G-quadruplexes or triplex structures are known. Ligands specific to the structures formed by disease-specific DNA and RNA repeat motifs have been shown to inhibit gene expression, alter gene splicing, or alter repeat instability (64, 65, 66, 67, 68, 69, 70, 71, 72, 73). Understanding the disease-specific DNA and RNA structures formed by the CANVAS-causing repeat motifs in *RFC1*, relative to the nonpathogenic motifs, is a beginning to opening such therapeutic paths.

## Experimental procedures

### Sample preparation

Oligonucleotides listed in Figure 1*B* were purchased from IDT and ACGT Corp. Concentrations were determined by recording the absorbance at 260 nm with molar extinction coefficients (Fig. 1*B*). Oligos were dissolved in 100 mM KCl, 10 mM Tris–HCl, and 0.1 mM EDTA (pH 7.4) unless otherwise stated. Samples were heated to 95 °C and allowed to cool to room temperature overnight.

### CD spectroscopy

CD spectra were measured using a Jasco J-810 spectropolarimeter, using 5 to 20 μM sequence in the stated buffer. For the CD spectra, samples were in a 0.1 cm pathlength cuvette, and spectra were measured over the wavelength range of 190 to 330 nm at a scan rate of 200 nm/min. Spectra shown are the average of three such spectra and were corrected against the buffer. CD data were converted to molar circular-dichroic absorption (Δε) based on strand concentration (C) using the equation:

(1) Δε=θ/(32980∗C∗l)

where *θ* is the measured ellipticity value (mdeg), *c* is the molar strand concentration (M), and *l* is the optical path of a used cuvette (cm).

### TDS

All experiments were performed on a Varian Cary 100 UV–Vis spectrophotometer using cuvettes with an optical path length of 1 cm in a Peltier multicell (6∗6) holder. Absorbance spectra were recorded in the 220 to 340 nm range, with a scan speed of 200 nm/min and a data interval of 1 nm. In all cases, the sample concentration was set at 0.005 mM, and they were dissolved in 10 mM Tris–HCl, 0.1 mM EDTA (pH 7.4) and 100 mM KCl or LiCl, or 6 mM potassium phosphate, 0.1 mM EDTA (pH 5.6), and 1.5 M LiCl or 25 mM MgCl_2_ when in need to lower the pH. TDS were normalized by dividing the raw data by its maximum positive value, where the highest positive peak gets a Y-value of +1.

### UV thermal melting

UV thermal melting experiments were monitored on a Varian Cary 100 spectrometer by recording changes in the UV absorption at either 260 nm or 295 nm using a 1 cm path length cuvette. Heating rate was adjusted to 1 °C/min with data intervals of 0.5 °C. *T*_m_ values were determined from dual baseline-corrected 1 to 0 normalized curves (1-native and 0-denatured forms) as temperatures at which half of the molecules were folded.

### Mixing curve

The single-stranded oligos of d(AAGGG)4 and d(TTCCC)4 were mixed together in weighed proportions to give d(AAGGG)4:d(TTCCC)4 mixtures with molar ratios of 80:20, 67:33, 60:40, 50:50, 40:60, 33:67, and 20:80. Together with samples of the individual strands, all were dissolved in 6 mM sodium phosphate buffer and 150 mM NaCl (pH 5.6) with a total DNA strand concentration of 5 μM. To overcome intrastrand pairing the samples were heated to 95 °C and allowed to cool overnight.

### Soret band shift

A prefolded d(AAGGG)4 with 50 μM concentration was titrated into a cuvette containing 7 μM of TMPyP4. Absorption spectra were collected with a Varian Cary 100 UV–Vis spectrophotometer from 350 nm to 520 nm using cuvettes with an optical path length of 1 cm. The concentration of free TMPyP4 was determined using an extinction coefficient of 2.26 × 10^5^ M^−1^ cm^−1^ and absorbance values at 424 nm. The titration was stopped when three successive additions of the sample resulted in no further shift of the Soret band.

All values were corrected for the dilution effect. The fraction (α) of TmPyP4 bond was determined as follows:

(2) $$\alpha =\left(\text{Abs}\;{\text{TMPyP}4}_{\text{free}}-{Abs}_{\text{mixture}}\right)/\left(\text{Abs}\;{\text{TMPyP}4}_{\text{free}}-{Abs}_{\text{bond}}\right)$$

where Abs TMPyP4_free_ is the absorbance of the free TMPyP4 in the absence of any added DNA, Abs_mixture_ is the absorbance at any point after the beginning of the addition of the DNA, and Abs_bond_ is the absorbance of fully bonded TmPyP4 measured at 424 nm (the Soret maxima for TMPyP4). Concentrations of free TMPyP4 ([TMPyP4]_free_) and the concentration of bound TMPyP4 ([TMPyP4]_bond_) were calculated as follows:

(3) $${\left[\text{TmPyP}4\right]}_{\text{free}}={\left[\text{TmPyP}4\right]}_{\text{corrected}}\left(1-\alpha \right)$$

(4) $${\left[TMPyP4\right]}_{\text{bond}}=\left({\left[TMPyP4\right]}_{\text{corrected}}-{\left[TMPyP4\right]}_{\text{free}}\right)$$

where [TMPyP4]_corrected_ represents the concentration of TMPyP4 corrected for the volume changes because of the titrated DNA.

The percentage of hypochromicity of the Soret band is calculated as follows:

(5) $$\%\text{hypochromicity}=\left[\left(\varepsilon_{\text{free}}-\varepsilon_{\text{bond}}\right)\right]*10\text{,}$$

$${\text{Where}\;\varepsilon }_{\text{bond}}=\text{Abs}\;{\text{TMPyP}4}_{\text{bond}}/{\left[\text{TMPyP}4\right]}_{\text{bond}}$$

## Data availability

The authors confirm that the data supporting the findings of this study are available within the article and its supporting information.

## Supporting information

This article contains supporting information.

## Conflict of interest

The authors declare that they have no conflicts of interest with the contents of this article.

## Supporting information

Fig. S1 Weighted average and observed CD spectra of (*A*) pathogenic d(AAGGG)4:d(CCCTT)4 mixture, (*B*) non-pathogenic d(AAAAG)4:d(CTTTT)4 mixture both at 0.33:0.67 molar proportion in 6 mM sodium phosphate buffer,150 mM NaCl (pH 5.6) at 25 °C which indicates the formation of a new structure (triplex) in the pathogenic but not non-pathogenic sequence.

Fig. S2 (*A*) TDS (*B*) Thermal Melting Profile of (*Blue*) d(AAAAG)4 in 6 mM Sodium Phosphate, 0.1 mM EDTA, 1.2 M LiCl (pH 5.6) and (*Orange*) d(AAAAG)4 in 10 mM Tris-HCl, 0.1 mM EDTA, 0.5 M MgCl_2_ (pH 7.4).
