# Supplementary figures and images for: Pathogenic CANVAS-causing but not nonpathogenic RFC1 DNA/RNA repeat motifs form quadruplex or triplex structures
Source: J Biol Chem. 2023 Sep 1;299(10):105202. doi: 10.1016/j.jbc.2023.105202 (PMC10563062; doi:10.1016/j.jbc.2023.105202)

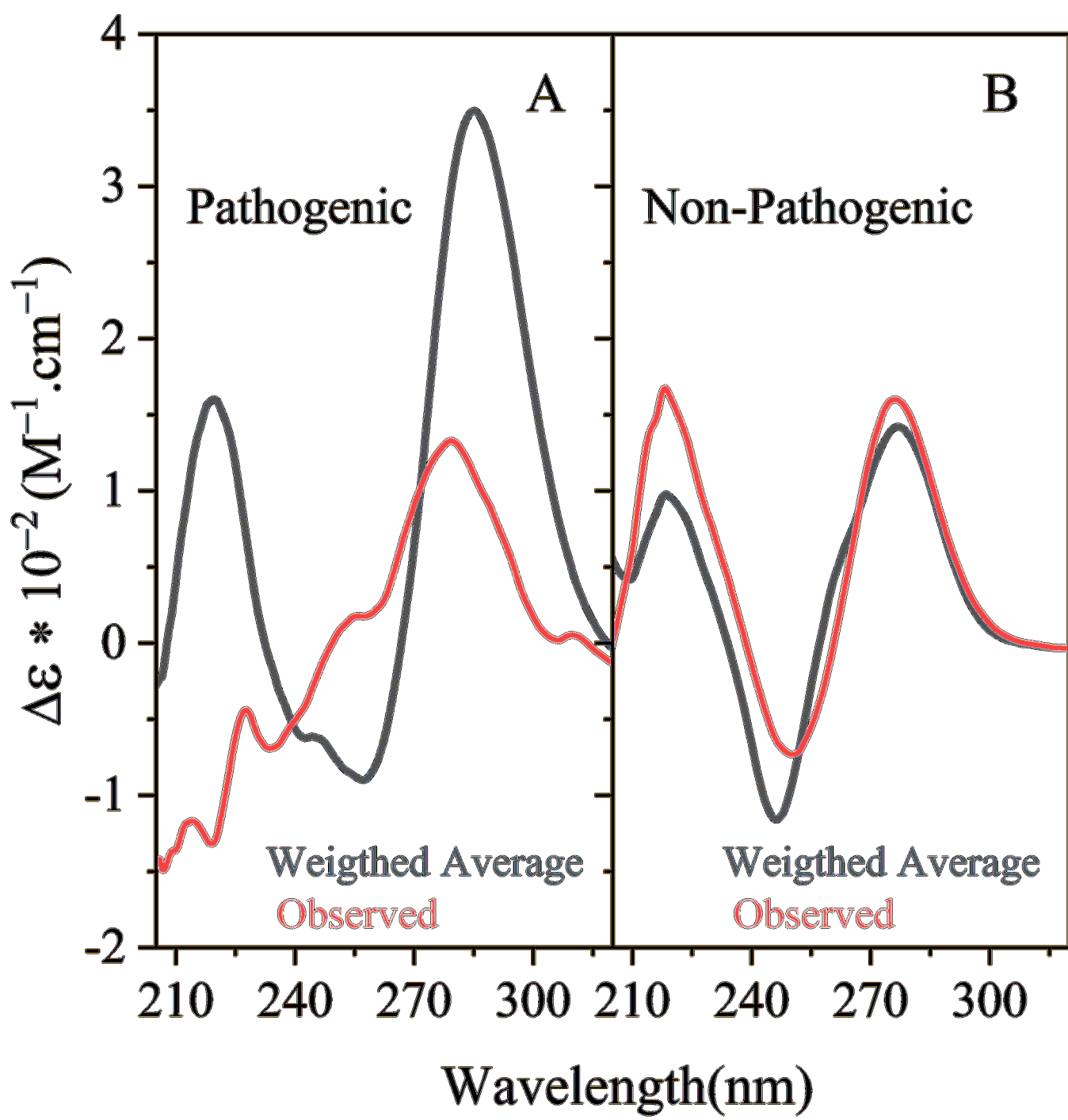

Supplement: Fig. S1 — Weighted average and observed CD spectra of (A) pathogenic d(AAGGG)4:d(CCCTT)4 mixture, (B) non-pathogenic d(AAAAG)4:d(CTTTT)4 mixture both at 0.33:0.67 molar proportion in 6 mM sodium phosphate buffer,150 mM NaCl (pH 5.6) at 25 °C which indicates the formation of a new structure (triplex) in the pathogenic but not non-pathogenic sequence. [file mmc1.pdf]

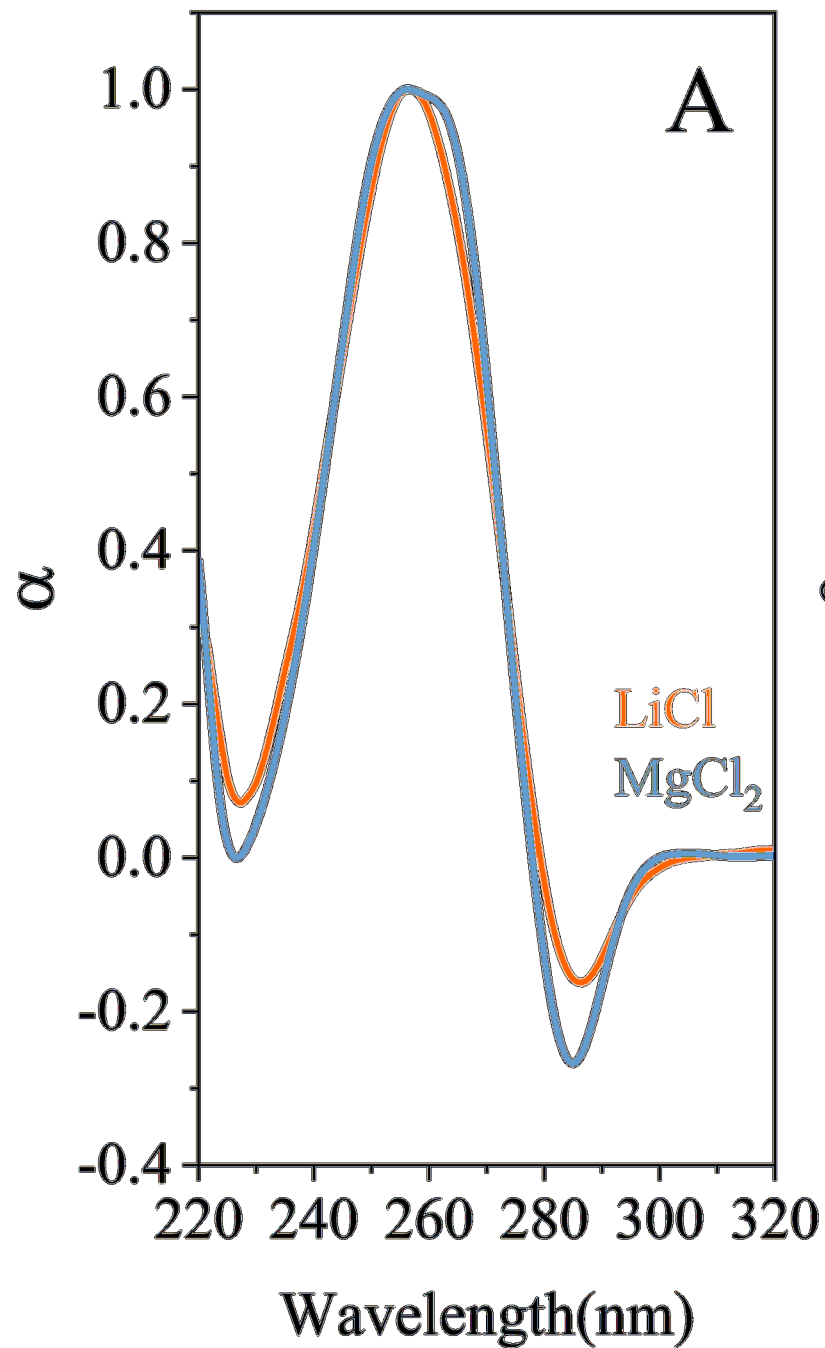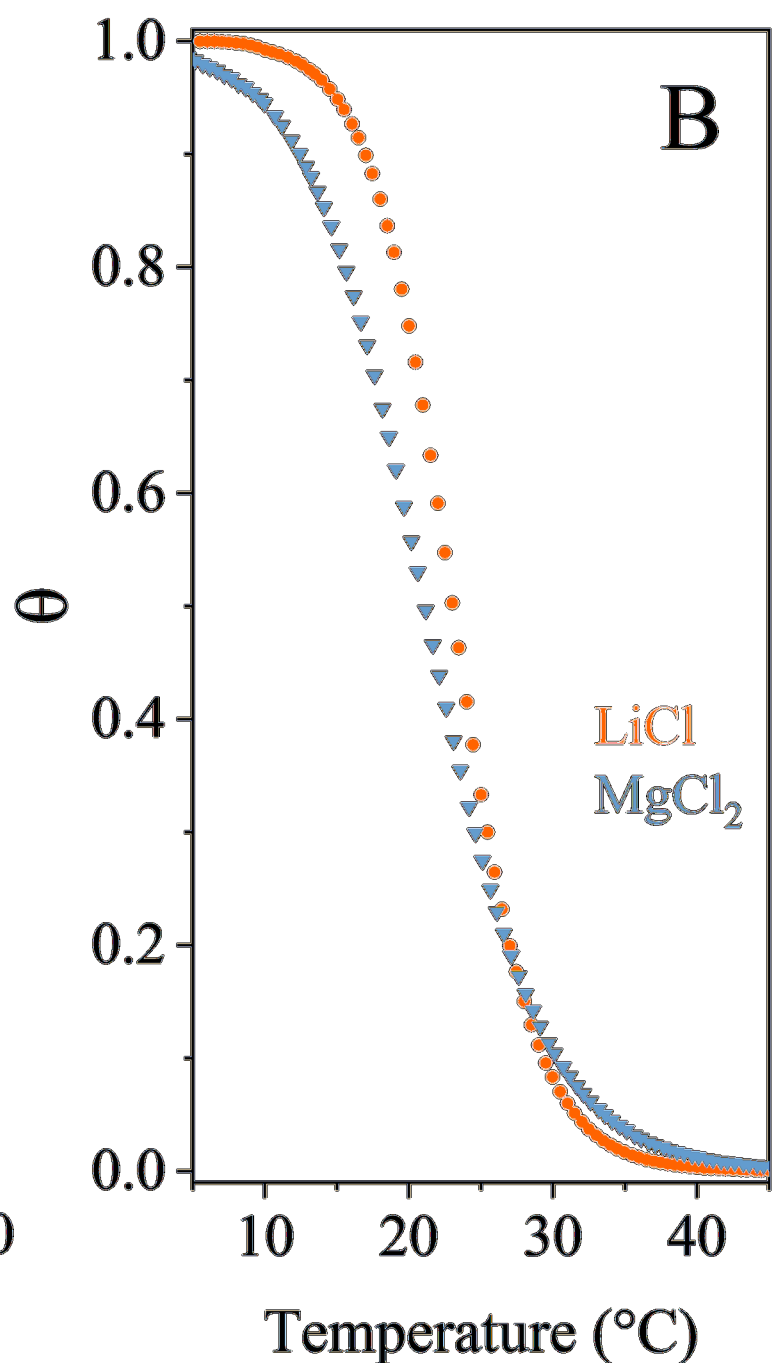

Supplement: Fig. S2 — (A) TDS (B) Thermal Melting Profile of (Blue) d(AAAAG)4 in 6 mM Sodium Phosphate, 0.1 mM EDTA, 1.2 M LiCl (pH 5.6) and (Orange) d(AAAAG)4 in 10 mM Tris-HCl, 0.1 mM EDTA, 0.5 M MgCl2 (pH 7.4). [file mmc2.pdf]
